# In silico identification of novel open reading frames in *Plasmodium falciparum* oocyte and salivary gland sporozoites using proteogenomics framework

**DOI:** 10.1186/s12936-021-03598-1

**Published:** 2021-02-05

**Authors:** Sophie Gunnarsson, Sudhakaran Prabakaran

**Affiliations:** Department of Genetics, University of Cambridge, Downing Site, Cambridge, CB2 3EH UK

## Abstract

**Background:**

*Plasmodium falciparum* causes the deadliest form of malaria, which remains one of the most prevalent infectious diseases. Unfortunately, the only licensed vaccine showed limited protection and resistance to anti-malarial drug is increasing, which can be largely attributed to the biological complexity of the parasite’s life cycle. The progression from one developmental stage to another in *P. falciparum* involves drastic changes in gene expressions, where its infectivity to human hosts varies greatly depending on the stage. Approaches to identify candidate genes that are responsible for the development of infectivity to human hosts typically involve differential gene expression analysis between stages. However, the detection may be limited to annotated proteins and open reading frames (ORFs) predicted using restrictive criteria.

**Methods:**

The above problem is particularly relevant for *P. falciparum*; whose genome annotation is relatively incomplete given its clinical significance. In this work, systems proteogenomics approach was used to address this challenge, as it allows computational detection of unannotated, novel Open Reading Frames (nORFs), which are neglected by conventional analyses. Two pairs of transcriptome/proteome were obtained from a previous study where one was collected in the mosquito-infectious oocyst sporozoite stage, and the other in the salivary gland sporozoite stage with human infectivity. They were then re-analysed using the proteogenomics framework to identify nORFs in each stage.

**Results:**

Translational products of nORFs that map to antisense, intergenic, intronic, 3′ UTR and 5′ UTR regions, as well as alternative reading frames of canonical proteins were detected. Some of these nORFs also showed differential expression between the two life cycle stages studied. Their regulatory roles were explored through further bioinformatics analyses including the expression regulation on the parent reference genes, in silico structure prediction, and gene ontology term enrichment analysis.

**Conclusion:**

The identification of nORFs in *P. falciparum* sporozoites highlights the biological complexity of the parasite. Although the analyses are solely computational, these results provide a starting point for further experimental validation of the existence and functional roles of these nORFs,

## Background

*Plasmodium falciparum* causes the deadliest form of malaria, which impacts over 200 million individuals and results in nearly 450,000 deaths each year, 60% of which are children aged under 5 years [1]. It belongs to the large Apicomplexa phylum of diverse eukaryotic intracellular parasites. In addition to malaria, this phylum also encompasses infectious agents of cryptosporidiosis and toxoplasmosis [2]. The latter is caused by *Toxoplasma gondii*, which is estimated to infect over 30% of the world population, and hence considered as one of the most successful parasites [3]. These diseases are not only life-threatening but also widespread and, therefore, represent a serious threat to public health.

Unfortunately, both vaccines and treatment options with drugs are limited due to the biological complexity of the Apicomplexan parasites [4]. Even for the well-studied *Plasmodium* species, there is only one vaccine targeted for malaria with relatively low efficacy [5, 6]. The complexity arises from the highly regulated life cycles that allow them to inhabit different hosts and intracellular niches [7]. To tackle this challenge, multi-omics data including genomic, transcriptomic, and proteomic datasets have been produced to understand the biological processes underlying infection and disease causation, which can potentially be targeted with drug design [8]. However, these studies are often conducted in silos and, therefore, their insights are limited due to the lack of consistency [9].

### Developmental cycle of *Plasmodium falciparum* and annotation challenge

Like many other Apicomplexan parasites [10], *P. falciparum* is specialized to infect two separate hosts—the human host, and the female mosquitoes of approximately 40 *Anopheles* species that can transmit the disease [11]. The developmental cycle starts with malaria-infected female *Anopheles* mosquito taking a blood meal, through which the sporozoites of *Plasmodium* parasites are transmitted from the mosquito’s salivary gland to the human host (Additional file 1: Figure S1). The sporozoites then travel to the liver and infect liver cells, allowing them to replicate and mature into schizonts. They then rupture and release merozoites, which infect red blood cells and is the stage that causes clinical manifestation of malaria. Some of the blood-stage parasites mature into sexual precursor cells, known as gametocytes, which are ingested by the *Anopheles* mosquito during a blood meal. The gametocytes are activated by environmental stimuli inside mosquito midgut and differentiate into gametes and fuse to form a zygote [12]. The zygotes further develop into oocysts, within which sporogony takes place and produces sporozoites to invade mosquito’s salivary gland, ready for next round of infection via a mosquito bite.

The progression from one developmental stage to another in *P. falciparum* involves drastic changes in gene expression [13], and infectivity of the parasites to human hosts can also vary greatly depending on the stage. For instance, while oocyst sporozoites are highly infectious for the mosquito salivary gland, they are non-infectious to mammalian hosts; on the contrary, salivary gland sporozoites exhibit specific infectivity for mammalian liver, but correspondingly loose infectivity for mosquito’s salivary gland [14]. Understanding the mechanism for this switch of infectivity is of great interest, because inhibiting it allows us to prevent infection before the symptomatic phase, which is where malaria intervention efforts using vaccines have been focused on [15].

Approaches to identify candidate genes that are responsible for the development of infectivity to mammalian hosts typically involve differential gene expression analysis between oocyst sporozoites and salivary gland sporozoites [16, 17]. However, although these studies have successfully identified genes and proteins that are upregulated in the mammalian-infectious stage, the detection may be limited to annotated proteins [9] and open reading frames (ORFs) predicted using restrictive criteria such as a minimum length of 100 codons [18].

This problem is particularly relevant for *P. falciparum*, whose genome annotation is relatively incomplete despite its clinical significance [19]. Even with the advancement of sequencing techniques and computational power, over 33% of predicted genes in the parasite genome remain functionally unannotated [20], which represent a great challenge for discovering vaccine candidates and therapeutic drugs. Furthermore, untranslated region such as 5′ and 3′ UTRs are also absent in the annotation, which have been shown to regulate protein expression in *P. falciparum* [21]. The lack of annotation despite community efforts can be attributed to several factors. As an *Apicomplexan* parasite, it is evolutionarily distant from other eukaryotic model organism where less than 25% of the protein sequences share significant similarity with those outside the phylum [22]. Even within the same phylum, many of the parasites have evolved pathways for cell invasion of their specific host and, therefore, only 12–34% of the protein-coding genes are shared by all apicomplexans [23]. Therefore, there is a pressing need for improving the genome annotation of *P. falciparum*.

To further complicate the annotation challenge, antisense transcription events have widely been discovered in *P. falciparum* and are thought to be important for gene expression regulation and parasite development between life cycle stages [24]. Of particular interests are the antisense transcripts reported in the *var* gene regions [25], which represent a gene family that encodes for roughly 60 different variants of *P. falciparum* erythrocyte membrane protein-1 (PfEMP1). PfEMP1 is a virulence factor that adhere to human receptors and hence enable the parasite to evade from the host immune system. To avoid recognition by antibodies, *P. falciparum* switches between the variant forms of PfEMP1 in a mutually exclusive manner [26]. However, the tightly controlled mechanisms underlying *var* gene transcriptional control remain largely unknown. It was recently shown that antisense long non-coding RNA (lncRNA) derived from intronic region of *var* can activate the corresponding *var* gene [27], which may shed light on *var* gene expression control. Unravelling the regulatory roles of these noncanonical regions, such as their coding potential, will require novel approaches that are specifically tailored to them.

### Proteogenomics and novel open reading frames (nORFs) discovery

**Fig. 1:**
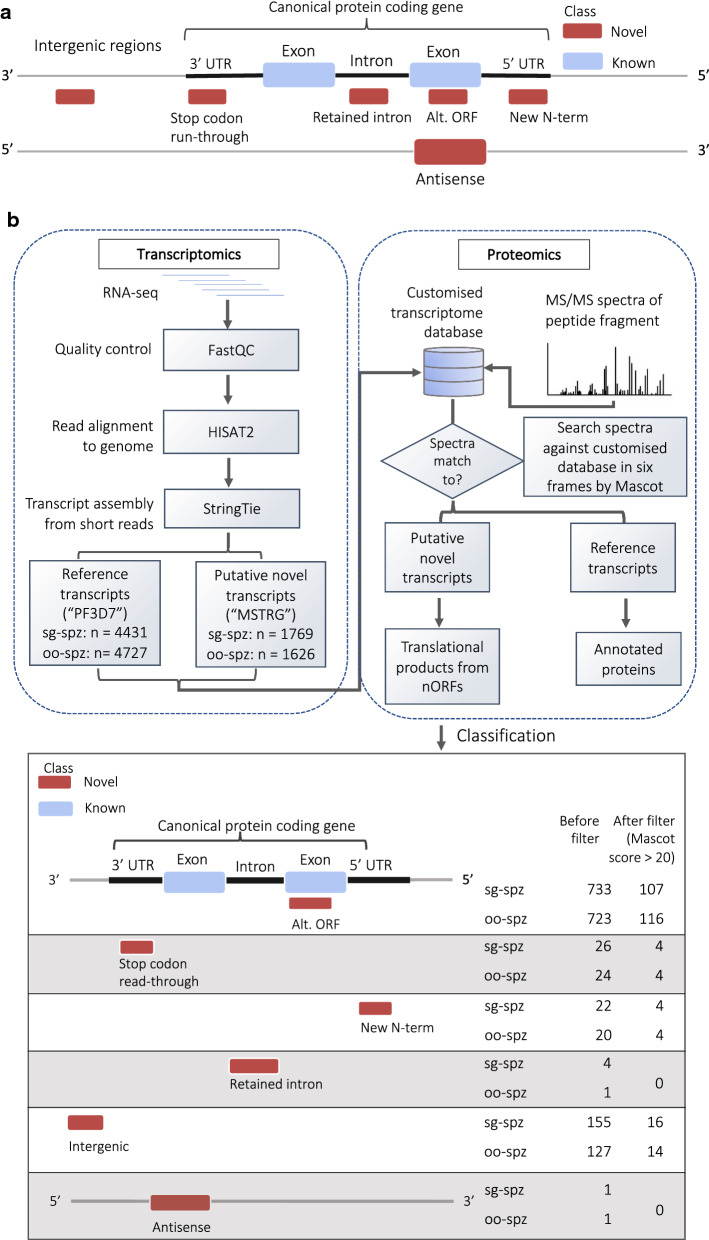
**a** The figure illustrates the nORF definitions used in this study. Translational products are quantified as peptide-spectral matches and classified into these five nORFs categories depending on where they map to the canonical gene. **b** Schematics of the proteogenomics analysis that was performed to identify translational products from novel open reading frames using transcriptomes and proteomes. oo-spz: oocyst sporozoite, sg-spz: salivary gland sporozoite from Lindner et al. [37]

One approach that can address the challenges above is proteogenomics, which combines the power of genomic, proteomic and transcriptomic data to improve genome annotation and the current understanding of protein expression (Additional file 1: Figure S2) [28]. The traditional proteomics analysis detects peptides by matching spectra from tandem mass spectrometry (MS/MS) against existing reference protein sequence database, which is prone to the assumption that the database contains all proteins in the genome of interest [29]. Especially for less well-annotated genomes, this assumption will have a more significant impact on peptide identification and downstream analysis like protein quantification. The peptide search in proteogenomic approach uses customized database instead, which can be generated by either six frame translation (6FT) of the genome, or by translation of the transcriptome [30]. It allows the detection of translation of nORFs that may otherwise be neglected, such as those from non-coding regions, including antisense transcripts, lncRNAs, intergenic and intronic sequences. The definitions of nORFs used in this study are as shown in Fig. 1a. Conversely, the proteomic data can provide peptide evidence for novel transcripts and RNA editing events, further improving the gene models. This approach can be iterative as more multi-omics data are being generated, and continuously improve genome annotation.

The use of proteogenomic approach has gained its popularity given the increasing amount of transcriptome data available thanks to next-generation sequencing [31, 32], as well as the improved accuracy and resolution in MS data for proteomics [33]. For the larger human genome, it has been used to identify novel coding regions in normal tissues and cancer cell lines, where a significantly larger number of novel peptides were discovered in the latter, indicating the biological importance of unannotated coding sequences [34]. The proteogenomic methods are particularly valuable for non-model organisms like *P. falciparum*, whose genome assembly and annotation tend to be incomplete, and protein database construction is difficult owing to the lack of close sequence relatives [35]. Previous successful examples include *Thermococcus gammatolerans*, an archaea extremophile whose genome was sequenced and annotated using proteogenomic analysis [36].

Here, proteogenomic analysis was performed using the transcriptomic and proteomic data obtained from the oocyst sporozoites and salivary gland sporozoites of *P. falciparum* used by a previous study [37]. nORFs were detected in both life cycle stages. Bioinformatics analyses suggest that these nORFs may be of functional importance with interesting properties and are, therefore, worth further experimental investigation.

## Methods

### Dataset

A literature review was conducted to identify the datasets suitable for proteogenomic analysis, which should consist of a pair of proteomes and transcriptomes from the same developmental stage. Thanks to the substantial research efforts devoted to *P. falciparum*, several transcriptome and proteome datasets have been deposited in the NCBI Sequence Read Archive (NCBI-SRA) and the PRIDE database, respectively [38]. However, it was difficult to find both transcriptomes and proteomes from the same stage, and some are not available in retrievable format. For instance, a study by Lasonder et al. [39] reported the transcriptomes and associated proteomes of female and male gametocytes, but the proteomic data was submitted to PlasmoDB [40], which could not be retrieved as raw data. Additionally, omics-data for *P. falciparum* are usually generated from different, independent studies, making the downstream analysis susceptible to batch effects.

As a result, this work focuses on two pairs of transcriptome/proteome generated by Lindner et al. [37]. The authors produced transcriptomic and proteomic data for oocyst sporozoites from wild-type *P. falciparum* parasites (NF54 strain) as well as salivary gland sporozoites, with three biological replicates for each sample type. The reasons for narrowing down to these datasets are threefold: firstly, the original analysis by Lindner et al. only considered annotated genes, leaving room for the discovery of novel ORFs. Secondly, the two life cycle stages analysed correspond to the mosquito-infectious and human-infectious stage, and therefore novel ORFs detected by proteogenomic analysis could help explain the development of human-specific infectivity. Finally, since the datasets were produced in the same laboratory, the conditions should presumably be similar and differential expression is mainly caused by biological differences between the developmental stages. The transcriptomic data was downloaded from the GEO database (Accession #GSM3109291, GSM3109292, GSM3109293 for oocysts sporozoites; # GSM3109294, GSM3109295, GSM3109296 for salivary gland sporozoites), and the proteomic data from PRIDE (Accession #PXD009726 for salivary gland sporozoites, #PXD009728 for oocysts sporozoites).

### Proteogenomic workflow

The novelty of proteogenomic analysis comes from matching the MS/MS spectra against a customized database constructed [28] based on the transcript information, instead of limiting the searches to known proteins as in the analysis by Lindner et al. [37] and in most proteomic analyses [41]. In this work, a customized database was constructed from the transcripts assembled from the HISAT2-StringTie pipeline as described by Pertea et al. [42] (Fig. 1b). However, one should be aware of the limitation of discovering novel ORFs using this approach, as it is just a computational prediction.

Briefly, the quality of RNA-seq data from oocyst sporozoites and salivary gland sporozoites were assessed using FastQC to check for contamination from sequences from other species, which is essential given that separating the parasite from its mosquito vector still represents a significant technical challenge [43]. The adapter sequences ligated to DNA fragments for library preparation were subsequently trimmed using Cutadapt [44]. The processed reads were then aligned to the reference genome for *P. falciparum* 3D7 strain retrieved from PlasmoDB ver.46 [40] using HISAT2 [45]. The BAM files with sequence alignment data were used to assemble transcripts using StringTie [46], which was guided by the reference annotation GFF file (PlasmoDB ver.46) and also allow the assembly of novel transcripts including splice variants. The assembled transcripts, therefore, include known transcripts from reference annotation with “PF3D7” as transcript ID prefix, and potentially novel transcripts with “MSTRG” prefix, which are constructed from reads that cannot be explained by reference transcripts. Compared to gene-level quantification where all reads are mapped to known genes, transcript-based approach taken by StringTie does not assume that genome annotation is complete and effectively expands the search space for matching MS/MS peptide spectra. The transcript nucleotide sequences were then extracted from the reference genome using BEDTools getfasta [47]. The constructed transcriptome database was then searched in six-frames for matching to MS/MS spectra by Mascot, a search engine that identifies protein using the MS data [48]. It matches all peptide spectra to the in silico translated proteins derived from the transcriptomic database “on the fly”, hence identifies which transcript is supported by peptide evidence. For each peptide-spectrum match (PSM), Mascot also computes a probability score which is higher for proteins with more peptides matched to it.

### Novel peptides classification

Since the known genes have been analysed thoroughly by Lindner et al. when they reported the transcriptomes and proteomes [37], only potentially novel transcripts with “MSTRG” prefix were considered in discovering nORFs that have been overlooked previously. Peptides that identify these MSTRG transcripts were subsequently classified (Fig. 1b) into different categories as defined above based on the position of matched peptides relative to the reference genes. Each category was classified independently as described below.

### Antisense

The first step to identify antisense peptides was to identify antisense transcripts from the pool of potentially novel MSTRG transcripts. Firstly, all the assembled transcripts were compared with reference transcripts using BEDtools intersect [47], using parameters that only return transcript that is on the complementary strand of the reference transcript that it overlaps with. Peptides that are matched to these antisense transcripts are then compared with the protein sequences from six frame translation using Transeq [49] to see which frame it originated from. Only peptides that are translated from frame 4 to 6 are considered translation evidence for antisense transcripts, because it could be degenerate peptides from reference genes due to six frame translation.

### Intergenic

The intergenic regions were extracted by using BEDtools complement [47] to subtract the genome from all annotated regions and return genome intervals with no genes identified. The MSTRG transcripts were mapped to these intervals with BEDtools intersect, returning transcripts that overlap completely are classified as intergenic. Intergenic transcripts with peptides from any frame that identified them are intergenic ORFs.

### Retained intron

MSTRG transcripts that contain the introns of overlapping reference genes are determined by GffCompare [50] that compares their intron–exon structures. The exons of these transcripts were subsequently intersected with the introns of reference genes to extract the retained intron region, which were six frame translated by Transeq and mapped by the peptides to see if they fall into the introns The intronic peptides were then checked manually to see if they are in-frame with the neighbouring exon on genome visualizer, Artemis [51]; if not, they are classified as AltORFs instead.

### 3′ and 5′ UTR

Unlike other well-characterized genomes, information about the untranslated regions of *P. falciparum* is relatively scarce [52], where the majority of research has been focussed on coding sequences only. Therefore, the first attempt to extract 3′ and 5′ UTRs by subtracting coding sequence from the genomic coordinates of mRNAs failed, because they are of equal lengths. BEDtools intersect was combined with length filtering to find MSTRG transcripts with complete overlap with a reference gene, and are also longer than the overlapped gene. These transcripts contain extended region on the 3′ and/or 5′ end outside coding sequence and regarded as UTRs accordingly. The UTRs were then translated into protein sequence to check if any peptides can confirm their translation. Similarly, peptides from UTRs need to be in-frame with the parent reference gene to be considered, otherwise moved to the AltORFs category.

### AltORF

Along with the out-of-frame peptides from retained intron and UTR classification, all peptides from MSTRG transcripts (excluding antisense transcripts) that overlap with known genes were also compared with the corresponding six frame translated coding sequences. The frame from which the peptides are translated can then be detected, and those from non-canonical frame, were classified as products of AltORFs. However, it is worth noting that almost half of these AltORF peptides map to frames that are antisense (frame 4, 5 and 6) to the canonical genes (341/732 peptides in oocyst sporozoites, and 379/733 peptides in salivary gland sporozoites), these peptides are categorized as AltORF products as they are not associated with antisense transcript.

### Differential expression analysis

To identify novel ORFs that may be involved in the development of infectivity to human hosts, differential expression analysis between the two stages studied was conducted at both transcript and protein levels. For RNA-seq data, DESeq 2 [53] was chosen to perform differential expression analysis, which is an R package available within the Bioconductor project [54]. This is because DESeq 2 is widely used by the community, including Lindner et al. that published the analysed dataset [37], and it has also been recently proved to have the best overall performance among 12 methods [55]. Since DESeq2 requires read counts as an input, while StringTie outputs coverage values for transcript abundance, these were first converted from coverage to counts for each transcript, using the formula *reads_per_transcript *= coverage * transcript_len/read_len with a python script (available at http://ccb.jhu.edu/software/stringtie/dl/prepDE.py). DESeq2 then normalizes the counts internally and compares them between oocyst sporozoites and salivary gland, producing statistics metrics including adjusted p-values and log-transformed fold changes. Transcripts with adjusted p-values < 0.1 were called differentially expressed between the two stages.

For protein differential expression analysis, the spectral count method was used for protein expression analysis that compares the peptide-spectrum matches (PSM) for each protein between the two stages, which has the highest reproducibility for label-free proteomics data [56]; it is then combined with the G test statistics [57] for computing p-values. However, in this study the MS/MS spectra were matched against a customized transcriptome database instead of annotated protein database, definition of a protein was, therefore, adjusted. Because the protein sequence of a translated transcript is different for each frame, an assumption was made that a given peptide can only identify one frame of the matched transcript being translated, and that a maximum of one protein can be translated from each frame of a transcript. As a result, the sum of PSMs that identify one frame of a transcript are the spectral counts for the protein product from that frame of the transcript.

Based on this assumption, the spectral counts were computed for each frame of each transcript (potential protein) with peptide evidence, and increase all of them by 1 to remove zero-values. These counts were subsequently normalized by first calculating the sum of all PSMs of both samples to identify the sample with smaller sum, where its PSMs were multiplied by the ratio of two PSM sums to minimize the background effect between samples. It was then possible to determine the differences in spectral counts of a protein between two stages by applying the G test of significance as follows:

$${\text{G = 2[C}}_{\text{oo}} { \ln }\left( {\frac{{{\text{C}}_{\text{oo}} }}{{ (\frac{{{\text{C}}_{\text{sg + }} {\text{C}}_{\text{oo}} }}{ 2} )}}} \right){\text{ + C}}_{\text{sg}} { \ln }\left( {\frac{{{\text{C}}_{\text{sg}} }}{{ (\frac{{{\text{C}}_{\text{sg + }} {\text{C}}_{\text{oo}} }}{ 2} )}}} \right) ]$$

where G is the G test static, *C*_*oo*_ and *C*_*sg*_ are the normalized spectral counts for a protein in oocyst sporozoite and salivary gland sporozoite respectively. A p-value was calculated as the probability that a *χ*^2^ distribution with 1 degree of freedom was more extreme than the G statistic for that protein. The Benjamini–Hochberg method was used to correct for false discovery rates from multiple hypothesis testing, where a protein needs to satisfy the criteria of both G-test and FDR < 5.0% to be called differentially expressed.

### Estimation of full-length ORF from peptide-spectral match

While differential expression analysis compares the expression level of one protein between samples, it is also interesting to compare the relative abundance of a protein with other proteins within and between samples. Therefore, the normalized spectral abundance factors (NSAF) were computed for each protein, which has been proved to provide reliable quantification [58]. For a given protein k, the spectral abundance factor (SAF) is calculated as the total PSMs (or spectral counts) that identifies the protein normalized by its length, and the NSAF is then the SAF normalized by total SAF values in the sample as shown below:

$$( {\text{NSAF)}}_{k} = \frac{{(SpC/Length)_{k} }}{{\mathop \sum \nolimits_{i = 1}^{N} (SpC/Length)_{i} }}$$

where (NSAF)_k_ is the NSAF value for protein k, SpC is spectral count and length is the protein length. This method adjusts for the protein length, because larger proteins tend to have higher probabilities of generating more PSMs; as well as the total protein abundance in one sample. However, for peptides that do not map to canonical coding sequences, the open reading frame in the mapped transcript from which they were translated needed to be estimated to obtain protein length. The estimated ORFs could also be used for downstream functional analysis.

Briefly, for all transcripts with peptide-spectrum matches, their spliced nucleotide sequences were first extracted and six frame translation was performed. Then, for each peptide-spectrum match, the frame of which the matched transcript it maps to was identified and the translated protein sequence was extracted. All possible open reading frames were subsequently determined from the protein sequence defined by the presence of start and stop codon, which is indicated by methionine and “*” respectively in the protein sequence. The matched peptide was then mapped to these possible ORFs and see if it matches to any of them. If not, the ORF will be defined as the sequence flanked by the start and stop codons closet to the matched peptide (Additional file 1: Figure S3). An R script was written to perform this task and can be found in https://github.com/PrabakaranGroup/nORFs-in-malaria.

### Gene ontology term and pathway enrichment analysis

All categories of nORFs analysed in this work except for intergenic ORFs have overlaps with a known gene. The sets of reference genes that overlapped with novel ORFs identified in the oocyst sporozoites and salivary gland sporozoites, respectively, were extracted and GO term and KEGG pathway enrichment analysis was performed on these genes using the Analyze tools on PlasmoDB [40]. InterProScan [59] was used instead to predict GO terms in novel ORFs.

### Structural prediction

For small proteins with less than 200 amino acids, the structures were predicted using an ab initio structure prediction tool QUARK [60], which has shown top-ranking performance in the Critical Assessment of Structure Prediction (CASP) experiments consistently [61]. Since template-free, ab initio methods work best on small proteins, QUARK has a size limit of 200 amino acids, and larger proteins were predicted using the template-based I-TASSER [62] instead.

## Results

### Identification of novel transcripts and peptides

Using the HISAT2-StringTie [42] workflow to align the RNA-seq reads to reference genome and assemble into full length transcripts, a total of unique 7844 transcripts were detected, with 4727 and 4431 canonical transcripts identified in oocyst sporozoite (oo-spz) and salivary gland sporozoite (sg-spz) stages, respectively, which are comparable with the results reported by Lindner et al. (3535 and 3575). The log transformed fold changes of the canonical transcripts also correlate reasonably well with their data (Additional file 1: Figure S4a), with a correlation coefficient of − 0.533, where the negative correlation is caused by the stages being compared (oo-spz:sg-spz vs sg-spz:oo-spz). An addition of 2045 transcripts were also identified, which are assembled from reads that could not be explained by canonical transcripts (with “MSTRG” transcript prefix). These potentially novel transcripts were then classified into different categories based on their comparison with the overlapped reference genes as described in “Methods” section (Fig. 1b). From the MSTRG transcripts, 780 and 790 transcripts were predicted to contain 3′ and 5′ UTR regions that are unseen in canonical transcripts along with 326 transcripts with retained introns. In addition, 41 antisense transcripts and 427 intergenic transcripts were detected. Identification of AltORFs requires translational evidence and are, therefore, not applicable. The high abundance of transcripts from the UTR and intergenic regions agrees with a recent study [63] that reported nearly 90% of the *P. falciparum* genome is being actively transcribed. Together, these results demonstrated the possibility of identifying nORFs in untranslated regions that could not have been possible using traditional methods, which might have biological functions given their transcriptional potential. The gene boundaries also need to be re-defined to take the regions outside coding sequence into consideration.

**Fig. 2:**
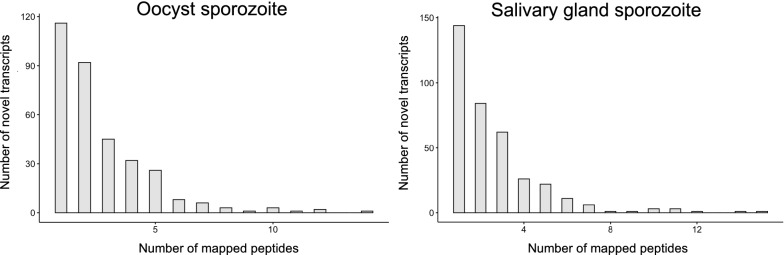
Distribution of number of unique peptides mapped to novel transcripts identified in oocyst sporozoite and salivary gland sporozoite

However, transcriptional evidence alone tends to be very noisy, especially for non-canonical transcripts that are constructed from de novo assembly [64]. With the availability of proteomic data, it was possible to use the peptide-spectrum matches (PSMs) from novel transcripts to identify their existence with much higher confidence. As shown in Fig. 2, most of the novel transcripts have one or two unique peptides mapped to them, with very rare cases of over ten peptides. This suggests that most of these novel transcripts tend to be translated in only one frame even with six-frame translation and the translated region probably covers a small segment of the transcript.

Furthermore, a relatively small fraction of novel transcripts has translation evidence from proteomic data, which emphasizes the need to confirm their existence experimentally. Less than 1/3 of the intergenic transcripts from the two stages have peptide spectral matches that can identify them (n = 130), although many of them are specific to either salivary gland or oocyst sporozoites (Table 1). Notably, despite many transcripts showed retained introns of canonical genes, only five of them have peptide evidence that supports the translation of these introns, indicating that the current intron–exon structures of coding sequences are likely to be accurate. Similarly, antisense peptides were detected at a very low level, with one PSM in each stage. Unlike other untranslated regions, slightly more novel peptides were detected to be translated from the 3’ and 5′ UTRs. One explanation is that they reflect the incomplete annotation of reference genes, or they could arise from stop-codon read-through [65] and translation of upstream ORFs respectively, where the latter has been shown to regulate the expression of downstream canonical ORFs [66]. However, despite the sparse evidence for translation of these novel transcripts, their regulatory roles on canonical proteins may be at RNA level only, especially antisense transcripts, whose RNA molecules were shown to interact with chromatin and regulate gene expression in *P. falciparum* [25].

**Table 1:** Number of novel transcripts identified from RNA-seq data and number of novel transcripts with translational evidence in oocyst and salivary gland sporozoites using the proteogenomic workflow

	Antisense	Intergenic	Retained intron	3′ UTR	5′ UTR	AltORF	
	41	427	326	780	790	/	Novel transcript
Oocyst sporozoite	1	64	1	22	18	261	Novel transcript with peptides
Salivary gland sporozoite	1	87	4	24	21	261	
Transcript common in both stages	0	21	0	7	8	132	
Total unique transcript	2	130	5	39	31	390	

Majority of the novel peptides belong to the alternative ORF (AltORF) category, which was defined as the translational products from a non-canonical reading frame of the overlapped coding sequence, including those that fall into introns and the 3′ and 5′ UTR regions of the transcript. AltORFs have previously been found in viruses, bacteriophages as well as humans [67], but their existence has not yet been reported in *P. falciparum*. This suggests that the proteome of *P. falciparum* may be much more complicated than previously thought, and the abundance of AltORFs suggests that the parasite may use it to expand the coding potential of existing genes in the compact genome. Although the functions of AltORFs remain largely unknown, it was thought that the translation of these non-canonical ORFs alone could provide a mechanism for expression control [68]. It would, therefore, be interesting to investigate if these novel peptides could explain the development of infectivity for human host in the parasite.

### ORF prediction and differential expression analysis

Although the identification of nORFs in this study is limited to computational prediction, it is worthwhile to investigate whether some of them are more interesting targets for experimental verification than others. To study the potential regulatory roles of the novel peptides, differential expression analysis was performed on their parent proteins between oocyst sporozoites and salivary gland sporozoites. However, unlike most canonical proteins whose amino acid sequences have been identified experimentally, novel peptides themselves do not provide information of the full-length novel proteins from which they were generated, they are only evidence that there are PSMs that identify a segment of the transcript. An attempt was made to predict the open reading frame based on where the peptides map to translated sequence of transcripts (see “Methods” section).

This approach was applied to both putative novel proteins and canonical proteins, where the latter have annotated protein lengths that allow one to verify the feasibility of estimating full-length ORFs from PSMs of six-frame translated transcripts. By searching the MS/MS spectra against the customized transcriptome database, 2901 and 2933 canonical proteins in oocyst sporozoite and salivary gland sporozoite were identified, respectively, as compared to 1432 and 2040 proteins identified by Lindner et al. [37]. The results for the protein-length normalized spectral abundance factors (SAF) for canonical proteins were then compared with those reported by Lindner et al. The protein abundances (in SAF) that were computed for canonical proteins in the samples from two stages correlated strongly with the published data (Additional file 1: Figure S4b), with correlation coefficients of 0.95 and 0.91 for oocyst sporozoite and salivary gland sporozoite, respectively, indicating that one could deduce the ORFs for novel peptides using this method (Additional file 1: Figure S3) as well.

**Fig. 3:**
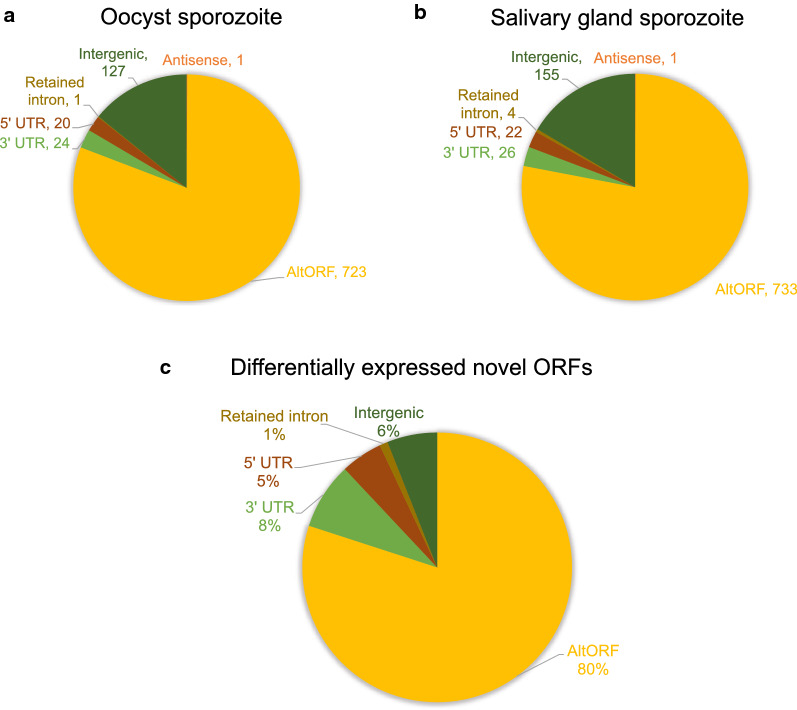
Distribution of nORF categories in total proteomes of oocyst sporozoite (**a**) and salivary gland sporozoite (**b**), as well as in differentially expressed nORFs (**c**)

After computing the PSMs for both novel and canonical proteins in two different stages, one could then proceed to identify the ones that were differentially expressed using G-test statistics. In total, 86 differentially expressed nORFs were observed, which share a similar distribution of nORF categories (classified in Fig. 1) with those present in the total proteomics data of salivary gland sporozoites and oocyst sporozoites (Fig. 3). One exception is the intergenic ORFs—while the intergenic peptides were the second most abundant category in both stages, only 6% of the differentially expressed nORFs are from the intergenic region.

### Antisense transcripts and peptides

Although only two unique antisense peptides were identified in this study, antisense transcripts have previously been shown to be incorporated into chromatin and consequently activate the virulence factor in *P. falciparum*, the *var* gene family [25]. It is, therefore, worth investigating further the regulatory roles of the antisense transcripts on their parent transcripts. The corresponding reference genes of the 41 antisense transcripts were first extracted and the transcript abundances in TPM (Transcripts Per Million) of both sense and antisense transcripts were calculated, and finally compared the TPM values using Pearson correlation. As a result, five antisense transcripts were shown to have significant correlation (p < 0.05) with the sense reference transcripts (Table 2), where three of them have positive correlation that suggests an activation mechanism. Interestingly, regardless of the transcript correlation, all reference genes with associated antisense transcripts are significantly downregulated at transcript level in salivary gland sporozoites. Especially for the two antisense transcripts that showed anticorrelated expression, one of the parent gene has both downregulated mRNA and protein expression, while the other is downregulated at mRNA level but upregulated at protein level. Notably, the MSTRG.401.1 transcript also has translational evidence, suggesting that the regulation by antisense transcripts could be mediated by their translational product. Overall, the actions of antisense transcripts observed are not uniform where they could potentially regulate the expression of their target genes not just by activation but also by repression, which has been well-established in mammalian genomes [69]. However, since the transcript level of the parent reference genes do not correlate well with protein expression, further experimental evidence is required to confirm the roles of these antisense transcripts.

**Table 2:** Correlation of TPM values between antisense transcripts and their associated reference transcripts across the two life cycle stages (oocyst and salivary gland sporozoites)

Associated reference transcript	Antisense transcript	Correlation coefficient	*p*-value	Reference transcript fold-change	Reference protein fold-change
PF3D7_0209600.1	MSTRG.182.1	0.984	0.000360	↓	ns
PF3D7_0103800.1	MSTRG.17.1	− 0.964	0.00187	↓	↓
PF3D7_0414500.1	MSTRG.618.1	0.947	0.00415	↓	ns
PF3D7_0314000.1	MSTRG.401.1	− 0.928	0.00758	↓	↑
PF3D7_1116000.1	MSTRG.2636.1	0.879	0.0211	↓	↓

↓ indicates that the fold change is negative in differential expression analysis and ↑ indicates positive fold-change. ns indicates that the reference transcript was not significantly differentially expressed

### GO term and pathway enrichment

**Fig. 4:**
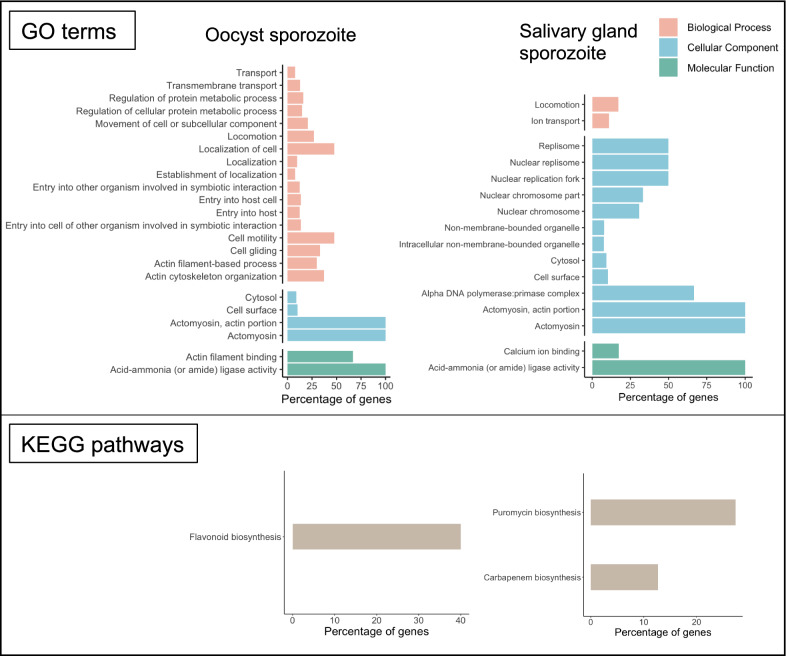
GO terms and KEGG pathways enrichment of the reference genes associated with novel peptides identified in oocyst sporozoite and salivary gland sporozoite. For GO terms enrichment the p-value cut-off was 0.01, and 0.02 for KEGG pathways. Percentage of genes means how many percents of the background genes (in *Plasmodium falciparum*) with a particular term are present in the gene set of interest

To investigate if nORFs could be involved in a particular function in *P. falciparum,* enrichment analysis was performed on the GO terms and KEGG pathways on their parent reference genes, because they might affect the functions of existing genes. As shown in Fig. 4, parent genes of the novel peptides identified from oocyst sporozoites and salivary gland sporozoites are associated with very different sets of known genes. With a stringent p-value cut-off of 0.01, the parent genes that overlap with the novel peptides in oocyst sporozoites have a clear enrichment in processes related to cell localization and movement, which is further supported by the 100% enrichment of the background genes in actomyosin. On the other hand, the gene set for salivary gland sporozoite is also enriched in the actomyosin structure, and distinctively enriched in replisome complex as well.

Since oocyst sporozoites need to migrate from mosquito midgut to salivary gland and await for injection into human host via a blood meal, motility is crucial for host invasion and achieved by the invasion machinery called glideosome, which is powered by the actomyosin system [70]. Given its importance to entry into host cells, attempt has been made to target the glideosome-associated proteins with small molecule as a potential approach to discover anti-malarial drugs [71]. Therefore, it shows that the identified nORFs are associated with important loci for infectivity and could serve as an extended reservoir to target for future drug design.

For KEGG pathway enrichment, a less stringent cut-off was chosen because otherwise no pathways could pass the filter, which still returned very few enriched pathways (Fig. 4). Interestingly, the gene set in salivary gland sporozoite is enriched in the pathway for the biosynthesis of an antibiotic, puromycin, where the genes involved in this pathway are all alpha/beta hydrolases. Of particular interest is the BEM 46-like protein, which was shown to modulate the development of sporozoites [72] and, therefore the associated novel peptides may play a role in the modulation as well.

### High quality peptide filtering and re-analysed differential expression

To obtain high quality novel peptides, a quality filter of Mascot score > 20 was applied on the proteomic datasets and subsequently performed differential expression analysis on the proteins of filtered peptides. Approximately half of the total peptides remained after applying the filter (Table 3), whereas for novel peptides the proportions are much lower, ranging from 14% to 20%. This suggests that compared with peptides from canonical proteins, novel peptides tend to have lower probabilities of being a real positive match. A total of 138 and 131 unique novel peptides remained in the oocyst and salivary gland sporozoite dataset respectively (Table 4) after the filtering, with AltORF being the most common category followed by intergenic, which follows a similar trend before filtering. Unfortunately, no peptides from antisense strand or retained intron passed the filter, which were very rare before filtering too.

**Table 3:** Percentage of novel peptides and total peptides passing the filter of Mascot score > 20. oo1, oo2, oo3 represent the three biological replicates from oocyst sporozoite and sg1, sg2, sg3 are the replicates from salivary gland sporozoite

Sample	Percentage of novel peptides passing the filter	Percentage of total peptides passing the filter
oo1	17.5	50.5
oo2	19.8	49.7
oo3	15.2	48.2
sg1	14.5	53.0
sg2	19.9	53.4
sg3	17.7	52.8

**Table 4:** Number of novel peptides from different categories that passed the quality filter of Mascot score > 20

	Number of unique novel peptides			
	5′ UTR	3′ UTR	AltORF	Intergenic
Oocyst sporozoite	4	4	116	14
Salivary gland sporozoite	4	4	107	16

The ORFs of the filtered novel peptides as described previously, and see which of them was differentially expressed. With a much smaller pool of high-quality peptides, only five unique novel ORFs showed differential expression between the two stages (Table 5), where AltORF is still the most common category, and one ORF from 3′ UTR and intergenic regions. Interestingly, four out of five DE nORFs showed opposite trend in transcript and protein expression, meaning that those that have positive fold-change at mRNA-level showed negative fold-change at protein level and vice versa. The poor correlation between mRNA and protein abundances are not uncommon, which is often caused by the regulatory mechanism governing transcripts or proteins, but sometimes by noise and experimental error as well [73]. Further experiments are required to confirm if it is the former.

**Table 5:** High quality (Mascot score > 20) novel ORFs identified by using filtered peptides only

Transcript ID	Frame	Category of nORF	PSM in oo-spz	PSM in sg-spz	Log ratio of PSMs	Log fold-change of transcripts	*p*-value
MSTRG.2270.1	1	3p UTR	3.32	16.00	2.27	− 1.74	0.0026
MSTRG.231.2	1	AltORF	28.75	1.00	− 4.85	11.57	0.0000
MSTRG.633.1	6	AltORF	22.12	2.00	− 3.47	ns	0.0000
MSTRG.4174.1	2	Intergenic	1.11	11.00	3.31	− 3.49	0.0022
MSTRG.4394.1	1	AltORF	1.11	15.00	3.76	− 3.34	0.0002

PSM values were normalized as described in “Methods” section

### Differentially expressed 3′ UTR

It was worth noting that the differentially expressed 3′ UTR (MSTRG.2270.1), despite showing higher expression in salivary gland sporozoites, the peptide spectrum-match that maps to the 3′-end of the canonical gene (gene identifier: PF3D7_1013400) was only present in oocyst sporozoites. This is in line with the RNA-seq data, where the 3′-end is clearly expressed in oocyst sporozoites but almost undetectable in salivary gland sporozoite. Even though there were only two PSMs from this nORF before normalization in oocyst sporozoites, the 3′ UTR was still captured at such low expression, it is unlikely that the absence of peptide from 3′ UTR in salivary gland sporozoites is due to chance or physical chemistry of the peptide ion. It is possible that the protein with 3′UTR is an isoform specific to the oocyst sporozoite stage.

To understand the function of the extended 3′UTR region, InterProScan [59] was used to analyse the sequence of this nORF. The results revealed that while the canonical gene PF3D7_1013400 has only a long stretch of “Non-cytoplasmic domain” identified, the extended 3′-end of 71 amino acids was predicted with multiple transmembrane helices.This is further confirmed by the prediction of transmembrane helices from TMHMM [74], which predicted two helices in the region after the canonical stop codon (Additional file 1 Figure S5). The inclusion of this transmembrane tail might also explain the low expression of this protein in oocyst sporozoites, because membrane proteins require specialised protocols to be fully solubilised in sample preparation [75].

Results from TMHMM also suggested that canonical protein is likely to be outside of the membrane, which coincides with the non-cytoplasmic domain detected by InterProScan. Therefore, the extended region in the 3′ end can potentially act as an anchor that tether the canonical protein to the membrane in oocyst sporozoites. When the parasite transitions into salivary gland sporozoite, the canonical stop codon is used and without expressing the 3′ end of transmembrane helices.

**Fig. 5:**
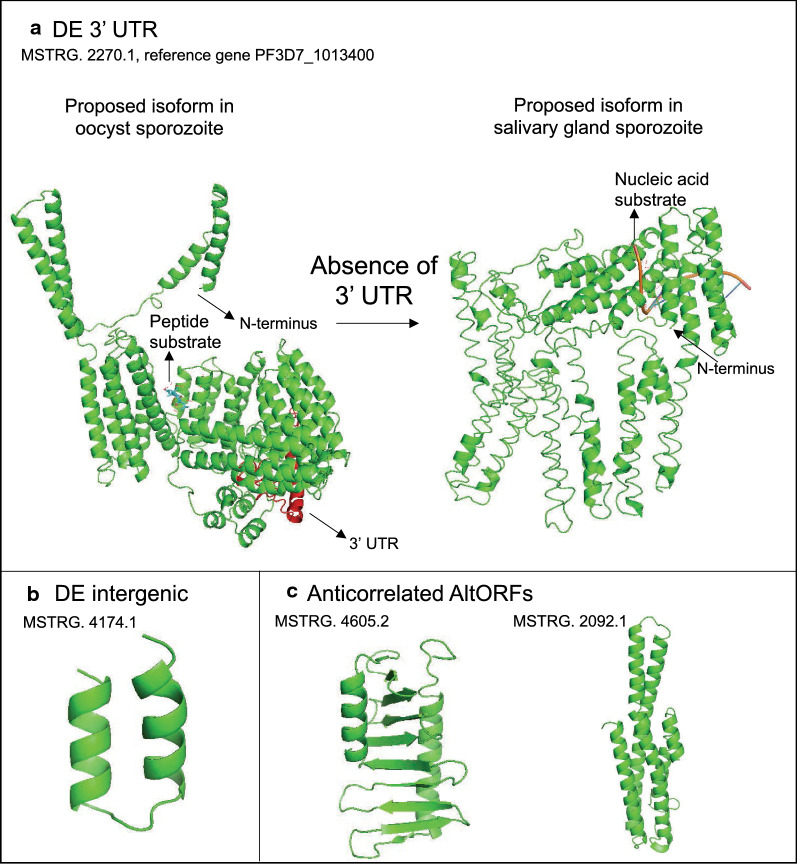
Predicted structures of **a** differentially expressed nORF (MSTRG.2270.1) with 3′UTR (3′ end coloured in red) of PF3D7_1013400 using I-TASSER; **b** differentially expressed intergenic nORF (MSTRG.4174.1) using QUARK; and **c** two AltORFs that have protein expression that was anticorrelated with the associated reference genes using QUARK

Since there is no function annotated for the parent gene of this 3′UTR other than it encodes a conserved protein, its structures with and without the 3′ extension were predicted using the template-based I-TASSER [62]. It appears that the isoform with extra helices at the 3′-end (Fig. 5a, highlighted in red) has a very different structure than the one without, where the former is predicted to have a more compact, ordered structure and likely to bind to a peptide substrate, while the latter contains a lot of disordered loops with nucleic acid substrate. Therefore, it is possible that the presence of extra helices allows the parent protein to tether to the membrane and adopt a more stable structure to bind to different substrates. When the parasite matures into salivary gland sporozoite, it expresses the canonical protein with no transmembrane helices, which may be released into the extracellular environment given its non-cytoplasmic domain and potentially involved in the host-parasite interaction upon infection.

### Intergenic ORF and transmembrane domains

Surprisingly, an intergenic nORF was also differentially expressed after the quality filtering. It is a short ORF with only 30 amino acids and unique to salivary gland sporozoites, identified by 10 raw PSMs (Table 5). InterProScan could not detect any functional domain possibly due to its short length and, therefore, its structure was modelled using an ab initio structure prediction tool QUARK [60], which can yield high-resolution structures for small proteins [76]. The predicted structure (Fig. 5b) is highly ordered with two short helices connected by a loop, suggesting that the intergenic ORF could form protein-like products too.

**Fig. 6:**
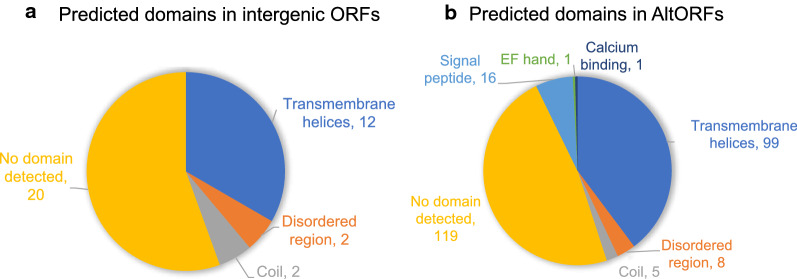
Predicted protein domains in high quality (Mascot score > 20) intergenic ORFs (**a**; total ORFs = 32) and AltORFs (**b**; total ORFs = 248) from InterProScan results

Intergenic ORFs with high-quality PSMs were then analysed to see if they have functional roles. Firstly, a BlastP search was performed on all the intergenic ORFs against the non-redundant protein sequence database, and no significant hit with expected value (E-value) smaller than 0.05 was returned, indicating that these ORFs share little or no sequence homology with known proteins. Interestingly, InterProScan detected many transmembrane domains in the intergenic ORFs from prediction by TMHMM (Fig. 6a), where 12 out of 34 were predicted with one or more transmembrane helices, and one of them was predicted with four helices. A similar scenario was observed in AltORFs (Fig. 6b) as well, where 99 out of 248 ORFs were predicted to contain transmembrane domain. Such abundance suggests that these ORFs may have biological functions in the membrane, which have been previously overlooked by the conventional annotation methods. Interestingly, this scenario has previously been observed in *Escherichia coli* as well [77], where over half of the novel small proteins (16–50 amino acids) identified in the intergenic region were predicted to have a transmembrane segment and also shown to co-fractionate with the membrane experimentally. Therefore, it is possible that this may be a common strategy adopted by organisms with small genomes to expand their proteome, although their exact functional roles still require experimental testing.

### Correlation of nORF expression with canonical gene

Finally, the correlation between the protein expression of high-quality nORFs and that of their associated canonical gene was tested. By computing the normalized spectral abundance factor (NSAF) values, it was possible to compare the expression across proteins and samples, which were used to perform Pearson Correlation analysis. In total, nORFs (Table 6) that showed significant expression correlation (p-values < 0.05) with the parent gene were identified, which are all AltORFs except for one 3′ UTR. It is worth noting that majority of them showed positive correlation, suggesting that either a positive regulation on the parent gene is a wide-spread scenario in nORFs, or that their expression is a by-product of the canonical translation event via mechanisms such as ribosome shifting [78].

**Table 6:** High quality novel ORFs that have significantly (*p*-value < 0.05) correlated protein expression (calculated in NSAF) with their associated reference genes

Transcript ID	Frame	Category of nORF	Reference gene	*p-*value	Correlation coefficient
MSTRG.4605.2	2	AltORF	PF3D7_1467600	0.0251	− 0.868
MSTRG.2092.1	6	AltORF	PF3D7_0927900	0.0425	− 0.827
MSTRG.1076.2	2	AltORF	PF3D7_0606600	0.0480	0.815
MSTRG.1556.1	2	AltORF	PF3D7_0728700	0.0457	0.820
MSTRG.3171.1	1	AltORF	PF3D7_1225600	0.0367	0.839
MSTRG.2151.1	6	AltORF	PF3D7_0934500	0.0149	0.898
MSTRG.2472.1	2	3p UTR	PF3D7_1033500	0.0134	0.904
MSTRG.2472.1	6	AltORF	PF3D7_1033500	0.0134	0.904
MSTRG.4173.1	2	AltORF	PF3D7_1417600	0.00838	0.924
MSTRG.370.1	1	AltORF	PF3D7_0311100	0.00560	0.938
MSTRG.3602.1	6	AltORF	PF3D7_1322400	0.00098	0.974
MSTRG.4140.1	1	AltORF	PF3D7_1414500	0.000685	0.979
MSTRG.2185.1	2	AltORF	PF3D7_1004300	0.000073	0.993

Interestingly, two AltORFs (MSTRG 4605.2 and MSTRG 2092.1) have anti-correlated expression with the parent gene, which are less likely to be a by-product and more likely have regulatory role on the expression of canonical proteins. Moreover, one of the parent genes, PF3D7_1467600 was significantly downregulated in salivary gland sporozoite, where the associated AltORF is uniquely present, suggesting that it may be involved in the downregulation mechanism. InterProScan did not find any functional domains other than transmembrane helices in the two AltORFs, which was common for novel ORFs as previously discussed. Therefore, an attempt was made to infer function from their three-dimensional structures by first predicting the structures using QUARK (Fig. 5c), and then submitting them to the structure-based function predictor COFACTOR [79]. Although there were no significant hits (C-score > 0.4) of the predicted Molecular Function GO terms for MSTRG 4605.2 (Table 7), MSTRG 2092.1 was predicted with nucleic acid binding function. Taken together, binding to the mRNA of canonical gene or to the chromosome could be a mechanism through which this AltORF regulates the expression of canonical proteins.

**Table 7:** Predicted molecular functions of the structures modelled by QUARK for MSTRG.4605.2 and MSTRG.2092.1, which showed negative correlation of protein expression with associated reference gene

MSTRG.4605.2		MSTRG.2092.1	
Molecular function	C-score	Molecular function	C-score
Phosphatidic acid binding	0.13	Nucleic acid binding	0.46
Phosphatidylinositol-4-phosphate binding	0.13	Phosphatase binding	0.40
Sterol transporter activity	0.13	Nucleic acid binding transcription factor activity	0.40
Oxysterol binding	0.13	Structural constituent of cytoskeleton	0.28
Phosphatidylinositol-4,5-bisphosphate binding	0.13	Catalytic activity	0.26

C-scores are confidence scores that range from 0 to 1, which higher score indicating a more confident prediction

## Discussion

Despite years of research efforts, malaria continues to be one of the most severe global health problems and affects millions of pregnant women and children in the African Regions [1]. This can be attributed to several factors including the rise of resistance to anti-malarial drugs [80] and the lack of a vaccine that has high efficacy [5]. It is, therefore, of great interest to discover new vaccine candidates and novel drug targets. Additionally, the genome annotation of *P. falciparum* remains relatively incomplete where a large portion of genes remains functionally unannotated and regulatory elements outside coding sequences are not included [20]. As an attempt to address these issues, proteogenomics analysis was performed to discover novel open reading frames that would not have been identified using conventional approaches and could enhance the current understanding in the parasite biology. The datasets analysed are the total transcriptomes and proteomes of oocyst sporozoites and salivary gland sporozoites [37], which correspond to the life-cycle stages that are mosquito-infectious and human-infectious, respectively, and the identified nORFs could, therefore, contribute to the understanding of the development of infectivity.

In this work, putative novel transcripts were classified based on how they map to the canonical genes and subsequently determined where their peptide-spectrum matches align to the translated sequence to identify novel peptides. A total of 1734 novel peptides were identified, where 269 of them passed the high-quality filter of Mascot score > 20. A method was also developed to predict the full-length open reading frame from peptides by finding the most suitable start and stop codons in the translated transcript, so that differential analysis could be performed for novel ORFs. The results for the length-normalized spectral abundance factors of canonical genes correlated well with those reported by Lindner et al. [37] (Additional file 1: Figure S4b), which were computed using annotated protein lengths, suggesting that the approach used is feasible.

By performing GO terms enrichment analysis on the canonical genes associated with the novel ORFs, it appears that they arise from functionally important loci that are critical to the parasite invasion and survival. While the gene set associated with novel ORFs identified in oocyst sporozoites was enriched in GO terms of motility and localization, the gene set for salivary gland sporozoites was more enriched in replisome-related GO terms (Fig. 4). More importantly, both gene sets showed significant enrichment in the genes that form the actomyosin complex, which is part of the parasite invasion machinery, also known as the glideosome. It is therefore possible that these novel ORFs play a role in host invasion and could serve as a drug target well, especially given that attempt has already been made to target glideosome with small molecules as a potential malaria therapy [71].

The functional roles of antisense transcripts have been a subject of debate where previous studies showed conflicting results—some suggest that they can activate transcription [25, 27] and others showed no effects [81, 82], where the former is achieved by epigenetic modifications and the latter is due to the lack of RNAi machineries. The presented results align with these observations where the levels of the 41 identified antisense transcripts do not have a consistent correlation with protein expression. Interestingly, some translation of the antisense transcripts was observed, suggesting that their regulatory roles could be performed by the translation product as well.

It was worth noting that while a lot of assembled transcripts have extended 3′/5′ ends and retained introns, very few peptides are mapped to these untranslated regions. Nevertheless, these UTRs tend to be regulatory elements that function at mRNA level and very little is known about their roles in transcriptional and translational regulation in the *Plasmodium* parasite [83], the results of this work could provide a starting point for further investigation. Furthermore, one high quality novel ORF from the 3′ UTR category was identified, which was differentially expressed between the two stages studied, and the peptide that maps to 3′ end was only observed in oocyst sporozoite despite low protein expression. The extra sequences provided by the 3′ extension are predicted to form a transmembrane helix and could affect the protein structure significantly, which may allow the protein to attach to the membrane and bind to different substrates. One possible explanation is that the parasite may choose to use different protein isoforms depending on the life cycle stage by skipping a stop codon and express the 3′ end, which could be an efficient mechanism to change infectivity for different hosts.

Finally, AltORFs and intergenic ORFs are surprisingly abundant, with AltORFs being the most common category in both stages as well as in differentially expressed novel ORFs. By correlating the nORF expression with their associated canonical genes, it was observed that most nORFs with significantly correlated expression are AltORFs showing positive correlation, except for two AltORFs that showed negative correlation. Structural analysis reveals that one of them is likely to form a protein structure that binds to nucleic acid, which provides a possible mechanism for this AltORF to regulate the expression of the associated gene by repressing its transcription or the translation of mRNA. On the other hand, intergenic ORFs were not only translated, some of them were even differentially expressed between oocyst and salivary gland sporozoites, suggesting that they might be involved in the stage-specific functions. An intriguing finding is that 12 out of 34 high-quality intergenic ORFs, and 99 out of 248 AltORFs were predicted with transmembrane domain, which may indicate that they have an important functional role in the membrane. Given that a similar scenario has been observed in *Escherichia coli*, non-canonical transmembrane ORFs might be common in organisms with small genomes to expand the functional proteome.

In summary, the novel ORFs in oocyst sporozoites and salivary gland sporozoites were identified through proteogenomics analysis, which allowed analysis on transcription and translation events outside the coding sequences that are annotated using conventional criteria. Combining analyses of differential expression, GO term enrichment and predicted structures, this work has shown that some of these nORFs may play a role in the parasite invasion and expression control. Therefore, they are interesting targets for further experimental validation on their existence and functional roles.

## Supplementary information

**Additional file 1. Figures S1 to S5.** Supplementary File for In silico identification of novel open reading frames in Plasmodium falciparum oocyte and salivary gland sporozoites using proteogenomics framework.

## Data Availability

All raw data in the main text or in the supplementary materials are from Lindner, S. E. et al. (2019) ‘Transcriptomics and proteomics reveal two waves of translational repression during the maturation of malaria parasite sporozoites’, *Nature Communications*, 10(1). 10.1038/s41467-019-12936-6.
