# Supplementary material for: In silico identification of novel open reading frames in Plasmodium falciparum oocyte and salivary gland sporozoites using proteogenomics framework
Source: Malar J. 2021 Feb 5;20:71. doi: 10.1186/s12936-021-03598-1 (PMC7866754; doi:10.1186/s12936-021-03598-1)
Supplement: Supplementary file 1 — Additional file 1. Figures S1 to S5. Supplementary File for In silico identification of novel open reading frames in Plasmodium falciparum oocyte and salivary gland sporozoites using proteogenomics framework. [file 12936_2021_3598_MOESM1_ESM.docx]

**In silico identification of novel open reading frames in *Plasmodium falciparum* oocyte and salivary gland sporozoites using proteogenomics framework**

Sophie Gunnarsson^1^, Sudhakaran Prabakaran^1*^

^1^Department of Genetics, University of Cambridge, Downing Site, CB2 3EH, UK

*Corresponding author

Email: [sp339@cam.ac.uk](mailto:sp339@cam.ac.uk)

**Supplementary Figure 1**

Liver

**Supplementary Figure 1.** Schematic illustration of the different developmental stages in *P. falciparum* involving mosquito vector and human host (Adapted from “DNA Repair Mechanisms and Their Biological Roles in the Malaria Parasite Plasmodium falciparum” by Lee et al., 2014 [84] `with modifications).

**Supplementary Figure 2**

**Supplementary Figure 2**. Concept of proteogenomic approach where genomics, transcriptomics, and proteomics are integrated to improve genome annotation and gene models (LC-MS/MS: Liquid Chromatography with tandem mass spectrometry)

**Supplementary Figure 3**


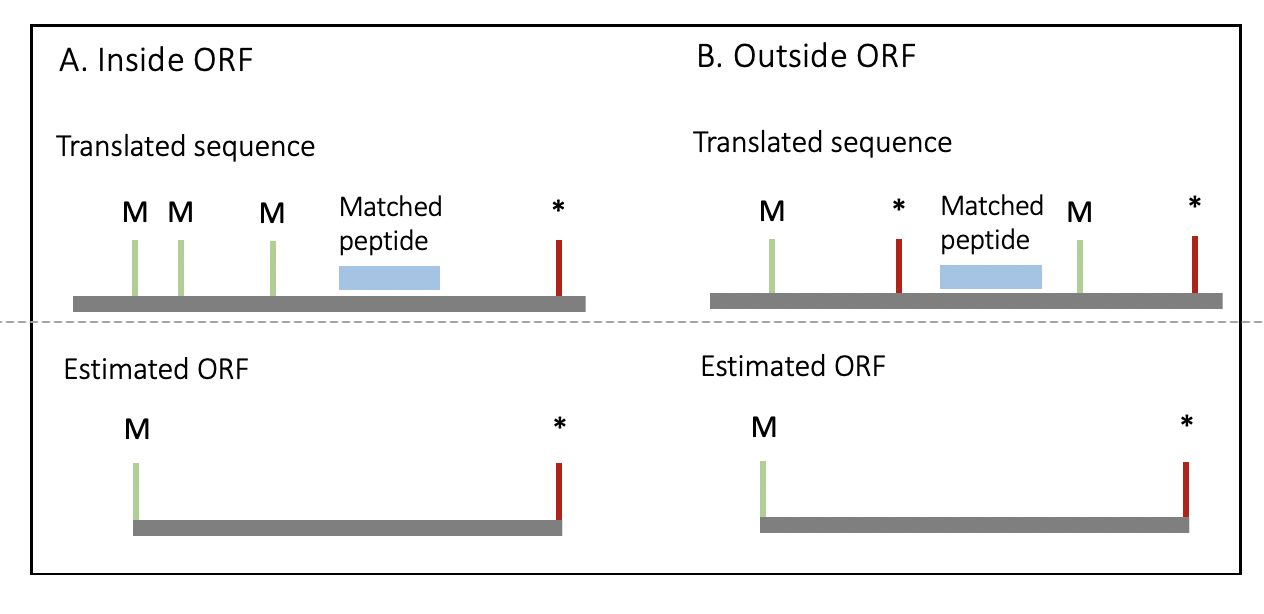


**Supplementary Figure 3.** Estimation of full-length ORF based on where peptides matched to a translated transcript using an in-house R script, where M is methionine translated from the start codon and * is translated from stop codon. Inside ORF: peptide mapped to an ORF defined by a start and stop codon; Outside ORF: peptide does not fall inside any ORFs in the translated transcript.

**Supplementary Figure 4**

**Supplementary Figure 4.** Correlation of mRNA log fold change (a.) and spectral abundance factors SAF (b.) between the reference values for canonical genes reported by Lindner et al., 2019 and the values calculated in this work.

**Supplementary Figure 5**

**Supplementary Figure 5.** Diagram showing the RNA-seq coverage of the 3’UTR of PF3D7_1013400 and the transmembrane region prediction by TMHMM. The peptide-spectrum match that maps to the 3’UTR was only found in oocyst sporozoite. oo-spz: oocyst sporozoite, sg-spz: salivary gland sporozoite.
